# COVID-19: Herausforderungen für forensische Therapie und Behandlungsqualität in Einrichtungen des Maßregelvollzuges

**DOI:** 10.1007/s00115-021-01102-3

**Published:** 2021-03-08

**Authors:** Peter Praus, Eva Biebinger, Harald Dreßing

**Affiliations:** 1 Klinik für Psychiatrie und Psychotherapie, J5, Zentralinstitut für Seelische Gesundheit, 68159 Mannheim, Deutschland; 2 Klinik für Forensische Psychiatrie, Pfalzklinikum für Neurologie und Psychiatrie, Weinstraße 100, 76889 Klingenmünster, Deutschland

## Hintergrund

Aus der weltweiten Verbreitung des neuartigen Coronavirus in den letzten Monaten mit ihren weitreichenden Auswirkungen auf die Gesundheit der Allgemeinbevölkerung und das öffentliche Leben erwachsen für die Behandlung und Versorgung psychiatrischer Patienten enorme Herausforderungen. Noch im Jahr 2014 war ca. jeder fünfte vollstationäre psychiatrische Behandlungsplatz im Maßregelvollzug angesiedelt [9]. Wenngleich seither keine weitere Veröffentlichung vergleichbarer systematisch erhobener Daten durch das Statistische Bundesamt mehr erfolgte, so ist angesichts der Belegungszahlen im Maßregelvollzug einzelner Bundesländer nicht davon auszugehen, dass sich die Situation heute grundlegend anders darstellt. Dies gemahnt im Hinblick auf die Sicherung der Behandlungsqualität und die Wahrung der Interessen und Rechte dieser auch in der psychiatrischen Fachwelt marginalisierten und stigmatisierten Patientengruppe zu besonderer Wachsamkeit und Vorsicht. Infolge einer rezessionsbedingten Verknappung wirtschaftlicher Ressourcen, die für Erhaltung und Ausbau öffentlich finanzierter forensisch-therapeutischer Behandlungseinrichtungen bereitgestellt werden, ist zu befürchten, dass in der Öffentlichkeit die „Sicherung“ psychisch erkrankter Rechtsbrecher gegenüber Behandlungsaufträgen an Gewicht gewinnen könnte [1]. Andererseits stellt eine zunehmende Nutzung digitaler Kommunikationsmittel im forensischen Kontext eine besondere Herausforderung dar. Für Patienten, die im Maßregelvollzug untergebracht sind, erweisen sich darüber hinaus gängige Empfehlungen für effektives Management von Pandemien, z. B. eine frühe Entlassung in häusliche Quarantäne, als wenig praktikabel. Beschränkungen des persönlichen Kontaktes zu Angehörigen und von Ausgängen können rasch de facto eine Einschränkung von Vollzugslockerungen darstellen; die betroffenen Patienten können in solchen Fällen geneigt sein, juristische Klärungen des Sachverhaltes herbeizuführen, z. B. indem sie ein Beschwerdeverfahren anstrengen.

Notwendigerweise mussten auch die im Kontext des Maßregelvollzuges zunächst ergriffenen Maßnahmen auf in früheren Pandemien erworbenem Erfahrungswissen basieren [4, 10], z. B. einer Ausweitung von Handhygiene und des Einsatzes von Desinfektionsmitteln, die Einhaltung von Mindestabständen und das Tragen von Gesichtsmasken. Es existieren bisher keine standardisierten Empfehlungen, wie mit einem derartigen Infektionsgeschehen im Maßregelvollzug umzugehen ist und inwieweit therapeutische und juristische Prozeduren (z. B. Anhörungen) einer effektiven Modifikation im Hinblick auf eine signifikante Senkung des Infektionsrisikos bedürften. Erst seit kurzer Zeit erfahren diese Fragestellungen zunehmende Beachtung und Diskussion [12]; gesichertes Erfahrungswissen ist jedoch noch kaum vorhanden.

## Erfahrungen mit COVID-19 im Maßregelvollzug

Bislang wurde lediglich ein Ausbruch von COVID-19-Erkrankungen in einem forensischen Krankenhaus in Toronto, Kanada, mit insgesamt 23 Infektionen (13 Patienten, 10 Mitarbeiter) innerhalb der Einrichtung und einem Todesfall unter den Patienten publiziert [11]. Die Autoren erklären, dass zusätzlich zu grundlegenden Hygienemaßnahmen und Abstandsregeln ein rigoroses Screening aller Personen, die die Einrichtung betraten, auf Anzeichen einer respiratorischen Infektion eingeführt wurde. Zudem wurden unbegleitete Ausgänge in die umgebenden Gemeinden zunächst reduziert und schließlich – im Bewusstsein, dass diese Maßnahme die Rehabilitationsmöglichkeiten der Patienten merklich beschneiden würde – gestoppt. Darüber hinaus wurden Gruppentherapien, wann immer Mindestabstände nicht eingehalten werden konnten, durch digitale Interventionen, Kleingruppen im Freien oder Einzeltherapien ersetzt. Zudem wurde die physische Anwesenheit der psychiatrischen Behandler in einem wöchentlichen Rotationssystem organisiert und um digitale Formate (Webex^TM^) erweitert, um direkte Kontakte mit den Patienten auf ein Minimum zu beschränken. Gemäß der Einschätzung der Autoren der Studie kommt im Hinblick auf das Management des berichteten und künftiger COVID-19-Ausbrüche innerhalb der Einrichtung der Implementierung einer Isolierstation und der konsequenten Testung aller Mitarbeiter und Patienten in betroffenen Abteilungen der Einrichtung eine essenzielle Bedeutung zu. Obgleich sich wahrscheinlich ein derartiges Beispiel aus einem anderen Rechtssystem nur schwer vollständig auf die hiesige Praxis übertragen lässt, sind einige grundsätzliche Fragen zum Management von COVD-19 im Kontext forensischer Therapie wahrscheinlich auch systemübergreifend relevant.

An der Klinik für Forensische Psychiatrie des Pfalzklinikums wurden insgesamt ähnliche Maßnahmen ergriffen: u. a. Entwicklung von Notfallplänen, Begrenzung therapeutischer Maßnahmen auf das Notwendigste unter Einhaltung der Hygienerichtlinien, Aussetzen stationsübergreifender Maßnahmen und Bildung stabiler „Kohorten“, regelmäßige Testung von Einzelpersonen und Abteilungen gemäß den Empfehlungen des Robert-Koch-Instituts, Reduzierung der Regelkommunikation bzw. Einsatz von Telefon- und Videokonferenzen (z. B. bei Anhörungen), Vorhalt von Einzelzimmern für Quarantänefälle, Besuchsverbot für Angehörige und Einschränkung von Vollzugslockerungen (z. B. Beurlaubungen und erweiterte Ausgänge). Bislang konnte im Zusammenhang mit den o. g. Maßnahmen ein Ausbruch von COVID-19 innerhalb der Klinik für Forensische Psychiatrie des Pfalzklinikums vermieden werden. Eine Übersicht über die getroffenen Maßnahmen ermöglicht Tab. 1. Diese können jedoch keine definitiven Handlungsanweisungen darstellen, da sie einerseits den baulichen und personellen Voraussetzungen der Kliniken sowie der jeweiligen Belegungssituation gerecht werden müssen. Andererseits werden sie beständig den Empfehlungen einer regelmäßig tagenden klinikumsübergreifenden Task-Force angepasst.

**Table 1:** 

Entwicklung von Notfallplänen für den Fall des Auftretens von COVID-19-Verdachtsfällen unter Patienten und Mitarbeitern der Klinik (z. B. Anordnung von Quarantänemaßnahmen für einzelne Stationen und das zugeordnete Personal, wiederholte Testung von Personal und Patienten der betroffenen Station)
Strikte Einhaltung von Hygieneregeln (Händedesinfektion, Abstandsregeln, Tragen von Mund-Nase-Bedeckungen bei Kontakten zu Mitarbeitern und Patienten etc.)
Begrenzung therapeutischer Maßnahmen auf das Notwendigste (unter Beachtung der Hygieneregeln)
Regelmäßige Testung von Einzelpersonen und Abteilungen gemäß den Empfehlungen des Robert-Koch-Instituts und in Abhängigkeit von der Intensität der Patientenkontakte (entweder per PCR- oder Antigenschnelltest)
Aussetzen stationsübergreifender Maßnahmen und Bildung von „Kohorten“ von Patienten und Mitarbeitern
Reduktion der Regelkommunikation/Einsatz von Telefon- und Videokonferenzen (z. B. Durchführung von Anhörungen als „Videokonferenz“)
Vorhalt von Einzelzimmern für Quarantänefälle (z. B. Neuaufnahmen mit unbekanntem Infektionsstatus, klinische Verdachtsfälle)
Besuchsverbot für Angehörige
Einschränkung von Vollzugslockerungen (z. B. Beurlaubungen, erweiterte Ausgänge)

## Konsequenzen für die Behandlung im Maßregelvollzug

Die Rolle der forensischen Psychiatrie im Spannungsfeld zwischen Behandlungsauftrag, Sicherung und individuellen Autonomie- und Entfaltungsansprüchen sowie Freiheitsrechten der Patienten erfährt nun durch zusätzliche Verpflichtungen im Rahmen von Infektionsschutz und Pandemiebekämpfung eine weitere Nuancierung. Im Speziellen müssen berechtigte Interessen der Öffentlichkeit und vulnerabler Patientengruppen innerhalb der Einrichtungen des Maßregelvollzuges an einem Schutz vor Infektionen gegenüber den Ansprüchen der Patienten auf Lockerungen und vollzugsöffnende Maßnahmen, die jedoch im Falle von Ausgängen und Beurlaubungen auch ein gesteigertes Ansteckungsrisiko bergen, sorgsam abgewogen werden. Auch Konflikte mit dem „Beschleunigungsgebot“ im Maßregelvollzug sind denkbar. Restriktionen müssen hierbei nicht nur vor den betroffenen Patienten vertreten, sondern auch vor den Strafvollstreckungskammern und Aufsichtsbehörden gerechtfertigt werden. Transparenz, Offenheit und klärungsorientierte Kommunikation sind hier unabdingbar. Zudem birgt es besondere Herausforderungen, einer gerichtlich untergebrachten Patientenklientel mit erhöhtem Risiko für aggressives Verhalten und Gewalt die konsequente Einhaltung von Abstandsregeln und Hygienemaßnahmen nahezubringen.

Informationstechnologien werden in der Begutachtung [2, 3] und Behandlung forensischer Patienten vermutlich an Bedeutung gewinnen [6], möglicherweise auch den Zugang zu forensischer Behandlung erleichtern und Behandlungsspielräume erweitern [5]. Kritisch abzuwägen sind demgegenüber das immanente Risiko einer erleichterten Kontaktaufnahme mit Tatopfern auf dem Wege neuer Informationstechnologien und eventuelle „Sicherheitslücken“ dieser Verfahren im Hinblick auf Vertraulichkeit und Schweigepflicht. Auch der vertrauliche Kontakt der Patienten zu ihren Verteidigern und die rechtssichere Durchführung von Anhörungen durch die Strafvollstreckungskammern sind hier von Bedeutung. Dies alles erfordert eine stetige, systematische Evaluation und Qualitätskontrolle dieser Maßnahmen im Hinblick auf die gewünschten Endpunkte, z. B. die Remission der zugrunde liegenden psychischen Störung, gesellschaftliche Rehabilitation oder – allgemeiner und umfassender formuliert – „Recovery“ [8]. Andererseits eröffnet sich durch die zunehmende Nutzung derartiger Technologien wahrscheinlich auch die Möglichkeit, eine Zunahme personell bedingter Engpässe in der psychotherapeutischen Versorgung forensischer Patienten [7] vor dem Hintergrund steigender Infektionszahlen und bei einer ggf. zunehmenden Zahl von Mitarbeitern forensischer Kliniken, die Quarantänemaßnahmen unterworfen sind, zu mildern.

## Fazit für die Praxis

- Allgemein akzeptierte Maßnahmen zur Pandemiebekämpfung, z. B. Kontaktbeschränkungen, können im forensischen Kontext mit begründeten Ansprüchen auf Vollzugslockerungen kollidieren.
- Aus der Sicht der Autoren dieses Beitrages haben sich die Bildung von stabilen Patienten- und Mitarbeiterkohorten, die Einführung von Screeningmaßnahmen (Temperaturmessung, Durchführung von PCR- und Antigenschnelltests) bei Mitarbeitern und Patienten, das strikte Beachten von Hygienemaßnahmen, z. B. die Einhaltung von Mindestabständen und das Tragen „medizinischer“ Mund-Nase-Bedeckungen (z. B. „chirurgische“ oder FFP2-Masken) sowie die Etablierung von Notfallplänen (z. B. Anordnung von Quarantänemaßnahmen für Stationen und deren Personal bei Verdachtsfällen) als zielführend erwiesen, um COVID-19-Infektionen unter den Patienten der Klinik zu verhindern.
- Neue Informationstechnologien werden im Maßregelvollzug künftig an Bedeutung gewinnen, bedürfen jedoch hinsichtlich Sicherheit und Effektivität noch der systematischen Evaluation.
- Transparente Regelungen und offene Kommunikation können die Akzeptanz von Maßnahmen zur Pandemiebekämpfung im Maßregelvollzug verbessern.
